# When Compliance Is Not Safety: The Regulatory Blind Spot in AI Companion Chatbots

**DOI:** 10.7759/cureus.105902

**Published:** 2026-03-26

**Authors:** Esteban Zavaleta-Monestel, Sebastián Arguedas-Chacón, Jeaustin Mora-Jiménez, Ricardo Millán González

**Affiliations:** 1 Pharmacy, Hospital Clinica Biblica, San Jose, CRI; 2 Research, Hospital Clínica Bíblica, San José, CRI; 3 Medicine, University of Costa Rica, San José, CRI

**Keywords:** adolescent mental health, artificial intelligence in psychiatry, radiosurgery artificial intelligence, regulatory & compliance, suicide prevention

## Abstract

Current regulations governing artificial intelligence (AI) companion chatbots primarily emphasize disclosure obligations, internal safety protocols, and documentation requirements. AI companion chatbots are systems designed to simulate ongoing social interaction through human-like responses, emotional continuity, and memory across repeated exchanges. However, procedural compliance does not necessarily ensure clinical safety, particularly when adolescents use these systems during emotional crises. Emerging evidence from independent audit studies of consumer chatbots and benchmarking evaluations of large language models (LLMs) for mental health tasks suggests that crisis-response performance can be inconsistent, including variable recognition of suicide risk, inconsistent escalation, limited referral quality, and instability across models and system updates. This editorial argues that safety should be defined by real-world behavioral performance rather than procedural safeguards alone and calls for independent crisis testing, transparent reporting, and longitudinal re-evaluation to better protect vulnerable users.

## Editorial

Current regulatory approaches to artificial intelligence (AI) companion chatbots emphasize disclosure obligations, internal safety protocols, and documentation requirements. As reflected in California's Senate Bill 243, companion chatbots are AI systems that engage users through natural language interaction and adaptive, human-like responses that may foster ongoing social or relational engagement. Consider the scenario these regulations are intended to address: a distressed adolescent, awake late at night, seeking support from an AI companion chatbot because it seems more available than parents, teachers, or clinicians. In such moments, safety is defined not by disclosures, reminders, or internal policy documents, but by the system's behavior, its responses, omissions, and capacity to mitigate or exacerbate risk. Nevertheless, current global governance of these systems prioritizes documentation over demonstrable safety in real-world distress situations [1,2].

AI companion chatbots are fundamentally distinct from task-oriented digital assistants. They are engineered to simulate empathy, maintain conversational memory, and foster emotional continuity. These attributes constitute the core appeal of such products. For children and adolescents, especially during vulnerable periods, these design elements exert significant psychological influence. Legislators have begun to recognize this impact. For example, California's Senate Bill 243 specifically regulates "companion chatbots", requiring disclosure of artificiality, internal suicide-prevention protocols, and periodic reminders to minors that the system is not human [2]. New York has implemented similar measures, requiring AI chatbots to detect and address suicidal ideation or self-harm and to provide periodic notice that users are not interacting with a human, with enforcement authority assigned to the state Attorney General [3,4]. Similar risk-based regulatory models in Europe emphasize classification, disclosure, and documentation requirements, yet rarely require independent demonstration of behavioral safety in crisis scenarios [5,6].

**Figure 1:**
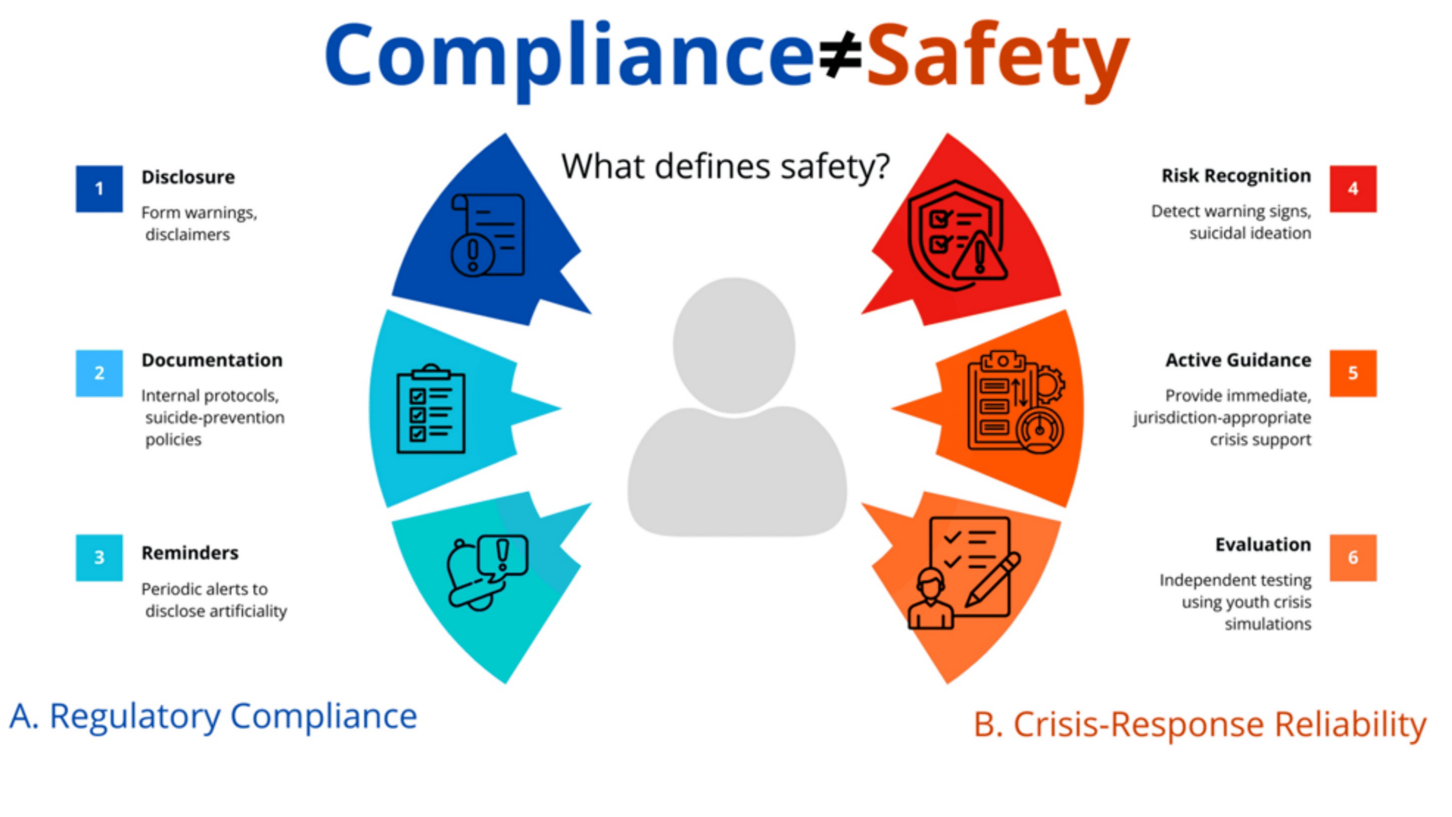
Procedural compliance versus crisis-response reliability in AI companion chatbots Conceptual comparison between regulatory safeguards and behavioral crisis-response performance in distressed adolescent users. This figure is a conceptual framework developed by the authors, informed by the regulatory and clinical literature cited [5], and was created using Canva (Canva Pty Ltd., Sydney, Australia).

These regulatory efforts are unified not by their ambition, but by their underlying logic. Safety is conceptualized as a procedural matter: disclosure, documentation, and assurances. While this approach is common in the initial phases of digital regulation, it is misaligned with clinical realities. In mental health care, the existence of a protocol does not constitute evidence of safety; actual outcomes do. This distinction between procedural compliance and behavioral reliability under crisis conditions is summarized in Figure 1, which contrasts regulatory safeguards with real-world crisis-response performance in distressed adolescent users [5].

Empirical evidence indicates that this gap is not merely theoretical. Independent audit studies show that consumer chatbots respond inconsistently to simulated adolescent crises involving suicidal ideation, sexual assault, and substance use, with fewer than half of responses rated as clinically appropriate, only 60% recognizing the need for escalation, and just 36% providing referrals to specific crisis resources. Performance was particularly poor among companion chatbots, which were substantially less likely than general-assistant chatbots to provide appropriate responses, recognize the need for escalation, or offer resource referrals [7].

Evaluations of large language models delivering mental health advice reveal similarly unstable performance across diagnostic tasks related to suicide ideation and depression, with marked variation between models and recurrent errors linked to symptom overlap, ambiguous language, limited clinical context, and defaulting to high-frequency categories when uncertain [8]. Notably, these deficiencies persist despite internal safeguards and may fluctuate with system updates, indicating that safety cannot be presumed stable over time.

To move beyond procedural reassurance, regulation must specify what counts as evidence of safety. At a minimum, this should include independent evaluation of companion chatbots using standardized youth crisis scenarios, red-team testing by external auditors with clinical and developmental expertise, longitudinal assessment of safety performance across major system updates, and verification that crisis responses and referrals are appropriate to the user's jurisdiction and language. Regulators should also require transparent reporting of crisis-response performance and re-certification after major system changes. These expectations are not unprecedented; they are consistent with established oversight for other high-risk interventions involving children, including pediatric medicines and medical devices, where external evaluation, post-market surveillance, and repeated safety reassessment are used to support safety and performance under real-world conditions rather than relying on internal policies alone [9-11]. 

This issue is particularly significant because adolescents may represent a vulnerable user group in emotionally charged interactions with AI systems. Companion chatbots are specifically designed to simulate attentiveness, memory, and emotional continuity, features that can strengthen perceived relational presence. Existing scholarship suggests that disclosure of artificiality does not reliably eliminate emotional influence during distress, nor does it prevent users from experiencing non-human systems as relationally meaningful. Consequently, regulatory frameworks that rely primarily on reminders of artificiality may rest on flawed assumptions about how young users experience these technologies [12].

The primary ethical concern is not that AI systems are imperfect, as human clinicians are also fallible. Rather, the concern is that current regulation tolerates imperfection without requiring evidence that these systems can reliably perform the functions most critical in high-risk situations: recognizing warning signs, providing appropriate and active crisis guidance, and undergoing standardized evaluation of those behaviors. In practice, this means demonstrating that emotionally responsive AI can identify suicide risk and other acute warning signals, avoid unsafe or minimizing responses, offer clear referral to appropriate human or emergency support, and do so consistently across repeated testing and major system updates. No youth mental health intervention would be considered acceptable if its safety rested solely on the existence of an untested protocol rather than evidence of reliable performance under stress. Yet this remains, in effect, the standard currently applied to emotionally responsive AI [7,12-14].

These implications are not limited to high-income contexts. Because current evidence already shows inconsistent crisis-response performance, limited referral quality, and sensitivity to language and clinical context, failures of localization may make crisis-related information and referrals less appropriate across jurisdictions and user settings [7,8]. This concern reinforces the need for independent evaluation across languages, locations, and system iterations rather than assuming that compliance measures will generalize safely across contexts [11].

Proponents of the current regulatory approach may contend that continuous behavioral auditing is impractical for rapidly evolving AI systems. However, feasibility has never exempted high-risk interventions from rigorous evaluation. In medicine and public health, systems that undergo frequent changes are still subject to post-market surveillance, repeated safety reassessment, and corrective action or withdrawal when safety cannot be maintained. Emotional AI systems designed to engage distressed children should not be held to lower standards solely because of their technological novelty [9,10].

Recent guidance has emphasized that artificial intelligence in healthcare should be governed in the public interest and with safeguards proportionate to the level of risk [11,13]. For emotionally responsive systems used by adolescents, this requires regulatory frameworks that go beyond disclosures, reminders, and internal documentation. Specifically, current frameworks should be revised to require independent behavioral audits based on standardized youth crisis scenarios, explicit failure thresholds for unsafe or non-escalating responses, transparent public reporting of crisis-response performance, and mandatory re-evaluation after major system updates [7,11].

These audits should assess whether systems reliably recognize suicide risk and other acute warning signs, provide active and context-appropriate crisis guidance, escalate appropriately to human or emergency support, and maintain acceptable performance across jurisdictions, languages, and successive product iterations [7,11]. Without such reforms, regulation risks becoming a form of safety theatre, in which visible procedural safeguards substitute for evidence that these systems behave safely under the conditions in which harm is most likely [10,11].

AI companion chatbots may eventually help address genuine gaps in mental health access. But innovation does not justify lowering the evidentiary standard for safety. As these systems increasingly intersect with adolescent mental health, they should be evaluated not as consumer novelties, but as behavioral interventions with real-world risk. Future research should therefore prioritize standardized adolescent crisis scenarios, validated measures of risk recognition and escalation, cross-jurisdictional and cross-linguistic evaluation, and longitudinal assessment of safety across repeated interactions and major system updates. For children and adolescents in distress, safety is not established by disclosures, reminders, or policy documents, but by how a system behaves in the moment of need. Until regulatory frameworks require evidence of that behavior, claims of safety, however well-intentioned, remain unproven.
